# The T-Line TL-200 system for continuous noninvasive blood pressure measurement in medical ICU patients

**DOI:** 10.1186/cc10820

**Published:** 2012-03-20

**Authors:** B Saugel, F Fassio, A Hapfelmeier, AS Meidert, RM Schmid, W Huber

**Affiliations:** 1 Klinikum Rechts der Isar, Technischen Universität München, Munich, Germany

## Introduction

The T-Line TL-200 (Tensys Medical Inc., San Diego, CA, USA) is a noninvasive arterial blood pressure (BP) monitoring system allowing continuous beat-to-beat monitoring of systolic arterial pressure (SAP), mean arterial pressure (MAP), and diastolic arterial pressure (DAP). It provides a real-time BP waveform like that obtained using an arterial catheter for BP monitoring. The aim of this study was to compare BP measurements obtained using the T-Line TL-200 system with simultaneous invasive BP measurements using a femoral arterial catheter in unselected critically ill medical patients.

## Methods

In 28 patients treated in a medical ICU of a German university hospital, BP values were simultaneously obtained using a femoral arterial catheter and the T-Line TL-200 device. All recorded data were included in the final analysis. For comparison of BP measurements, Bland-Altman analysis accounting for repeated measurements was performed.

## Results

A total of 76,826 pairs of BP measurements (each consisting of SAP, MAP, and DAP) were analyzed. For MAP, Bland-Altman analysis revealed a mean difference of +0.47 mmHg (95% limits of agreement: -16.53 to +17.46 mmHg). For SAP and DAP, the bias and 95% limits of agreement were -9.01 mmHg (-37.47 to +19.45 mmHg) and +5.22 mmHg (-13.50 to +23.94 mmHg), respectively.

## Conclusion

The T-Line TL-200 system allows determination of MAP with a satisfactory agreement when compared to invasive assessment of MAP using a femoral arterial catheter in unselected critically ill medical patients. Higher mean differences and 95% limits of agreement for SAP and DAP measurements might be explainable by limited comparability of central (femoral) and peripheral (radial) SAP and DAP measurements.

